# (Re)shaping Amazon labour struggles on both sides of the Atlantic: the power dynamics in Germany and the US amidst the pandemic

**DOI:** 10.1177/10242589221149496

**Published:** 2023-01-12

**Authors:** Sarrah Kassem

**Affiliations:** University of Tübingen, Germany

**Keywords:** Amazon, COVID-19, agency, strikes, unions

## Abstract

With the initial context of COVID-19 fuelling Amazon’s exponential growth, this article investigates how the pandemic (re)defined labour struggles, i.e., cultivating labour’s structural, associational and institutional powers in two case study countries, Germany and the US. By analysing these power resources in its two largest markets, I argue that Amazon’s structural conditions by which it organises its warehouse labour, which predate the pandemic, have continued to act as obstacles to collective labour action. While in Germany, ver.di continues to mobilise its workplace power but has been unable to get Amazon to sign a collective agreement, the pandemic triggered unprecedented workplace mobilisations and the pursuit of associational power in the US, albeit with varying outcomes. Despite their different industrial relations systems and labour struggles, these two cases highlight the key role of shop-floor organising to put pressure on Amazon, while Amazon’s continued rejection of unions as negotiating partners further underlines the importance of regulating Amazon’s union-busting tactics.

## Résumé

Dans un contexte où la COVID-19 a alimenté la croissance exponentielle d'Amazon, cet article examine comment la pandémie a (re)défini les luttes syndicales, en soulignant son impact sur les pouvoirs structurels, associatifs et institutionnels des travailleurs dans les deux pays étudiés, à savoir l'Allemagne et les États-Unis. En analysant ces ressources de pouvoir sur les deux plus importants marchés d'Amazon, l'auteur fait valoir que les modalités structurelles de l'organisation de sa main-d'œuvre par Amazon dans les entrepôts d'Amazon, qui sont antérieures à la pandémie, ont continué à faire obstacle à l'action collective des travailleurs. En Allemagne, le syndicat unifié des services (ver.di) continue de mobiliser ses forces sur le lieu de travail sans parvenir à faire signer une convention collective à Amazon. Aux États-Unis, la pandémie a entraîné des mouvements de mobilisation sans précédent sur les lieux de travail, à la recherche d'un pouvoir associatif, mais avec des résultats inégaux. Malgré leurs différences en termes de systèmes de relations industrielles et de luttes syndicales, ces deux cas mettent en évidence le rôle clé de la mobilisation des travailleurs à la base pour faire pression sur Amazon ; quant au fait qu'Amazon continue de récuser les syndicats en tant que partenaires de négociation, il souligne l'importance de réglementer les tactiques antisyndicales de cette entreprise.

## Zusammenfassung

Der Ausgangskontext für diesen Artikel ist das exponentielle Wachstum von Amazon infolge der COVID-19-Pandemie. Die Autorin untersucht, wie die Pandemie Arbeitskämpfe (neu) definiert hat, d. h. wie sie die strukturelle Macht, Organisationsmacht und institutionelle Macht der Arbeitnehmer:innen in zwei Ländern ihrer Fallstudie, in Deutschland und den USA, verändert hat. Anhand einer Analyse dieser Machtressourcen in den beiden größten Märkten von Amazon weist die Autorin nach, dass Amazons strukturelle Bedingungen, die gegen die Organisierung der Belegschaften in seinen Versandzentren bereits vor der Pandemie vorhanden waren, weiterhin als Hindernis für kollektive Aktionen der Arbeitnehmer:innen wirken. In Deutschland mobilisiert die Gewerkschaft ver.di weiterhin ihre betriebliche Machtbasis, konnte Amazon aber bisher nicht zur Unterzeichnung eines Tarifvertrags bewegen. In den USA hat die Pandemie zu einer ungeahnten Mobilisierungswelle in den Betrieben und zum weiteren Streben nach verbandlicher Macht geführt, allerdings mit unterschiedlichen Ergebnissen. Trotz der unterschiedlichen Systeme von Arbeitsbeziehungen und der unterschiedlichen Arbeitskampfsituationen in den beiden Ländern zeigen diese beiden Fallstudien die Schlüsselrolle auf, die der Organisierung in den Betrieben zukommt, um Druck gegenüber Amazon aufzubauen, während Amazons beharrliche Weigerung, Gewerkschaften als Verhandlungspartner anzuerkennen, weiter unterstreicht, wie wichtig eine Regulierung der gewerkschaftsfeindlichen Taktiken des Konzerns wäre.

## Introduction

Even before COVID-19, Amazon monopolised e-commerce and experienced exponential growth in many countries of the Global North. Moreover, it has been increasingly expanding in the Global South. Though Amazon, like many other platforms, is now struggling to sustain that growth and resorting to hiring freezes and lay-offs (Weise, 2022), the pandemic years 2020–2021 were characterised by surging orders. This was a period initially marked by border closures, brick-and-mortar shutdowns and job losses in some sectors of the (in)formal economy (see Bateman and Ross, 2021; Webb et al., 2020), while at the same time fuelling e-commerce. Amazon saw its capital and profits growing exponentially (BBC, 2021), prompting it to expand its workforce to power its growing infrastructure (Statista, 2021). In response to the spread of coronavirus, Amazon pointed to US$11.5bn invested in 150 health and safety measures (Amazon, 2022b), though warehouse workers initially reported a lack of PPE, no transparency over case numbers, and no possibility to socially distance (Kassem, 2020; O’Brien, 2022; Paul, 2020). Workers mobilised (trans)nationally around their health and safety concerns, with actions ranging from walkouts in the Global North and Global South to transnational coordinated actions during work peaks like Black Friday or the *Make Amazon Pay* campaign (Alimahomed-Wilson and Reese, 2021; Kassem, 2022; Vgontzas, 2021). These actions took place against a backdrop of public support and discourse around ‘essential labour’ during the pandemic, when Amazon warehouse workers were regarded as ‘frontline workers’ (Amnesty International, 2020) and even named ‘heroes’ by Amazon (Amazon, 2020; Vgontzas, 2021).

The interest in Amazon, both by researchers and unions, was sparked by the first industrial action in 2013 at an Amazon warehouse in Germany, an interest that grew in line with Amazon’s expansion and labour unrest. These mobilisations were in reaction to working conditions characterised by digital Taylorist division of labour into their smallest subtasks, organised and managed in the most efficient way (Delfanti, 2021). Amazon workers are not situated in production lines, as they do not produce items, but circulate them. I refer to these therefore as circulation lines (Kassem, 2023). Researchers have highlighted how Amazon sees its workers and warehouses as interchangeable. While it can easily replace its manual labour through pitting permanent and temporary workers against each other (Apicella, 2021; Barthel, 2019; Owczarek and Chełstowska, 2018), this logic is extended to its decentralised network of warehouses marked by no clear choke points (Barthel, 2019). Alongside investigating these structural conditions and their implications for undermining collective efforts to mobilise and unionise workers (Boewe and Schulten, 2019, 2020; Vgontzas, 2020), scholars have engaged with national cases to grasp the motivations of labour to resist and organise, examining such crucial elements as contract status, class-consciousness, desire for co-determination, and perceptions of Amazon’s working conditions (Apicella, 2021; Apicella and Hildebrandt, 2019). Given Amazon’s transnational nature, labour struggles had first to be contextualised within their national industrial relations settings before determining how to coordinate transnational action (Boewe and Schulten, 2019, 2020; Cattero and D’Onoforio, 2018; Transnational Social Strike, 2019).

As capital-labour relations are spatially and temporally bound, it is important to investigate Amazon in relation to the context of the pandemic and examine its implications for labour struggles at Amazon – a corporation that grew exponentially during the pandemic and whose workers played a key role in that growth. Workers organised around rising pandemic-related health and safety concerns (Kassem, 2022). With this in mind and building on the above-mentioned research, I ask: To what extent has the context of COVID-19 (re)defined labour struggles, i.e., cultivating labour’s structural, associational and institutional powers? To answer this question, I look at two countries, Germany and the US. As its two largest markets, they have the greatest potential to put pressure on Amazon, despite their substantially different industrial relations settings. I argue that Amazon’s structural conditions designed to fragment the workforce in and between warehouses, along with its anti-union position, continued to act as obstacles during the pandemic, undermining labour’s struggle across different industrial relations settings. The German services union ver.di continued its mobilisations but was unable to generate sufficient pressure through its workplace and associational powers to get Amazon to sign a collective bargaining agreement, ver.di’s decade-long goal. By contrast, the US case illustrates precisely how the pandemic’s context acted as a catalyst for both unprecedented workplace mobilisations and the pursuit of associational power through unionisation – though the latter was not always successful.

Divided into three parts and informed by critical political economy, this article starts by sketching labour’s agency in its struggle over interconnected structural, associational, and institutional power resources, examining *inter alia* the importance of focusing on Germany and the US. In the second part, I analyse the different labour struggles over these power resources during the pandemic, first in Germany and then in the US, placing them in their larger contexts. Contrasting these two cases demonstrates that, despite their different industrial relations settings and increased potential during the pandemic, Amazon was able to dodge negotiations with its workers through its undermining structural conditions and continued rejection of unions as negotiating partners. While it is therefore of key importance for labour to continue its efforts to mobilise and generate solidarity at shop-floor level, thereby boosting its power resources to put pressure on Amazon, it is also crucial to garner further political support to regulate and limit the company’s anti-union activities. Considering Amazon’s role as a trendsetter on the labour market (Delfanti, 2021; Kassem, 2023) and how it expanded its wealth, workforce and infrastructure during the pandemic, this analysis offers further insights for labour struggles beyond Amazon.

## Agency and labour’s interacting power resources

As capital develops and evolves alongside wider political-economic, societal and technological conditions (Harvey, 2010; Marx, 1977), Amazon has been one of the platforms instrumentalising the Internet and investing heavily in expansion over the last three decades and exponentially in recent years (UNI Global Union, 2020). Given that capital-labour relations are constantly in flux and intertwined (Bieler, 2018), this article focuses on the workers powering its warehouses, examining their agency through the interdependent and co-evolving structural, associational and institutional powers of their labour struggle. Contributing to the general scholarship on the power resources of platform workers (Barthel, 2019; Joyce et al., 2022; Kassem, 2023; Vandaele, 2018, 2020), this article contextualises these in relation to Amazon warehouse workers during the pandemic.

As capitalism is founded on its organisation of society into class relations, labour’s position within it is key to determining its ability to leverage capital for its own interests through structural power (Wright, 2000). Labour has two forms of structural power: marketplace power and workplace power. Relating to labour’s bargaining power on the labour market, the former is determined by such general factors as what the market regards as scarce skills, unemployment levels and dependence on wages as income. Workplace power is more specific, being defined as the possibilities for workers to instrumentalise their strategic position within their industry to disrupt capital’s activities, for instance by striking at key nodes of (trans)national value chains (Silver, 2003). Capital navigates the political-economic terrain to its advantage to weaken such efforts, for instance through the (re)location and (re)organisation of labour, or through (re)producing certain trends such as the further deregulation and precarisation of the labour market (Bispinck and Schulten, 2011; Gumbrell-McCormick, 2011; Silver, 2003). It is thus a continuous co-evolving process by which capital organises labour, thereby prompting a response from labour which may impact this relationship, reflecting Silver’s words ‘*where capital goes, labour-capital conflict follows shortly*’ (Silver, 2014: 50, emphasis in original). Given the interest in mobilisations of workplace power during COVID-19, this article focuses on contextualising the complex and not so straightforward terrain of *organised efforts*, including industrial action, walkouts and strikes.

Dependent on the context in which rights, actions and industrial relations determine employment relations (Hyman, 2005), workplace power can be further tied to associational power. This refers to labour’s collective organisation and association to pursue its interests, ranging from works councils to unions. Unions have historically been instrumental in securing and strengthening labour rights through their different power resources (Schmalz et al., 2018; Silver, 2003; Wright, 2000). Associational power is also linked to workplace power, as workers may feel more encouraged to participate in disruptions when protected by a union. At the same time these very disruptions can be opportunities for workers to not just mobilise, but also unionise. The mobilisation of workers in both workplace and associational terms is further determined by racialised and gendered divisions of labour, as class, gender, and racial subjectivities and realities are closely interlinked (Rose, 1997). They can in turn shape and reshape power resources in different ways. While capital can instrumentalise them to fragment groups of workers, they can also be a base for solidarity built on shared material experiences and interests (Bohrer, 2019; Hyman, 2011). In its attempts to undermine the structural power of workers, capital may also resort to restructuring – and thereby weakening associational power – to avoid losing control and having to negotiate with labour. Capital may, therefore, seek to ‘delegitimize existing trade union organizations and labor parties in the eyes of many workers by making it increasingly difficult for these organizations to deliver benefits to their members’ (Silver, 2003: 14). Given Amazon’s union-busting reputation, it is important to shed further light on the terrain that unions and workers need to navigate to collectively organise.

As associational and structural power cannot be dissociated from political-economic conditions and industrial relations, workers may cultivate, where possible, their institutional power to further improve their working conditions. Mobilising their workplace and associational power can aid them in engaging in negotiations with capital to achieve certain concessions and compromises (Schmalz and Dörre, 2014; Schmalz et al., 2018). Institutional power can be understood as the embodiment of workplace co-determination through works councils achievements and collective bargaining agreements with unions. Wright highlights how works councils, and in my view institutional power more generally, may not only be in labour’s interest but may also ‘serve employer interests in a variety of ways’, such as getting labour to accept certain working conditions with a view to increasing productivity and profits (Wright, 2000: 996). Amazon is one of the many employers seemingly unwilling to recognise unions as negotiating partners for collective agreements unless legally forced to do so, as seen in the sector-wide obligatory agreements in France and the exceptional negotiated agreement in Castel San Giovanni, Italy (Boewe and Schulten, 2019; Cattero and D’Onoforio, 2018). Accordingly, unions’ ability to put pressure on capital relates to cultivating and combining structural and associational power. At the same time, gains in institutional power, through for instance higher wages, can increase labour’s structural power and marketplace leverage.

Examining and contrasting different trajectories of the co-evolving power resources of Amazon warehouse workers can be helpful in grasping future possibilities to strengthen these both locally and nationally, and in turn transnationally. Though mobilisations have unfolded across the Global North and Global South, this article focuses on Germany and the US, two countries that experienced mobilisations of workplace power during the pandemic and constitute Amazon’s key markets in terms of net sales (the US ranked first, followed by Germany (Statista, 2022)). In the US, Amazon has become the second largest private sector employer (after Walmart), with its 950,000 US workers accounting for a sizeable share of its 1.3 million global workforce at the end of 2020. To put this figure into perspective, one in every 135 US workers works for Amazon (Reuter, 2021). On the other side of the Atlantic, Amazon defines itself as a ‘major European employer’ with 20,000 permanent logistics employees in Germany working in 20 of Amazon’s over 60 European logistics centres (Amazon, 2022a, 2022d). These statistics however lack transparency, as they take no account of the temporary and seasonal workers upon whom Amazon depends during its Black Friday and Christmas peaks. They also take no account of cross-border workers, such as those from Poland and the Czech Republic in Germany (Apicella, 2021; Boewe and Schulten, 2020) or those from Mexico in the US (Gurley, 2022).

While Germany and the US shared relatively greater potential to put pressure on Amazon through their workplace power mobilisations during the COVID-19 pandemic, Amazon was able to undermine these labour struggles, despite their different national contexts and industrial relations. With a workplace power dating back to the first strike in 2013, marking the longest record for Amazon labour mobilisations (Vgontzas, 2020), ver.di has a degree of associational and institutional power. It has been, however, unable to push Amazon into a collective agreement, even in a national industrial relations context that centres on co-determination. In contrast, the US workers demonstrate some of the newest organised efforts to mobilise and unionise and thus grow associational power closely related to the COVID-19 context – though Amazon undermines these through its union-busting efforts. A successful union drive would legally oblige Amazon to recognise the union and negotiate with it, carrying implications in terms of both associational and institutional power. These cases are thus interesting to compare in order to grasp how Amazon can continue to undermine labour struggles even at a moment where workers hold potentially high leverage, and how it can do so across their different political-economic and industrial relations contexts. Investigating these different labour struggles can be telling of repercussions and possibilities for its workforces to grow their power resources (trans)nationally, as they too navigate Amazon’s generally undermining structural conditions (Barthel, 2019; Vgontzas, 2020) and larger anti-union stance (Apicella, 2021).

This research is informed by qualitative analysis of online material (newspaper articles, scholarly work, accessible statistics, interviews and reports) and fieldwork conducted between 2018 and 2021. The latter included participating in union meetings in Germany and interviewing warehouse workers prior to the pandemic. I sporadically stayed in touch with some of them during the pandemic and recently interviewed a further worker in autumn 2022. I also participated in UNI Global Union’s transnational bi-annual (digital) Amazon Alliance meetings in 2018–2021 where unions from across the globe discussed ongoing developments. Considering the lack of transparency when it comes to union membership at Amazon and the fact that disclosing such figures may impact their leverage vis-à-vis Amazon, this article focuses on a general understanding of these labour struggles through their co-evolving power resources within their different contexts during the pandemic.

## Examining labour struggles on both sides of the Atlantic

Amazon operates an ever-growing decentralised network of logistics centres, generally without any of its warehouses or workers holding key strategic positions (Barthel, 2019; Vgontzas, 2020, 2021). These warehouses are characterised by a division of manual labour, with workers assigned to repetitive tasks along its circulation lines such as stowing, picking and packing items. Amazon locates its warehouses near to such infrastructures as airports and motorways in regions marked by higher unemployment and racialised and gendered labour markets. These points, along with Amazon offering wages and benefits above statutory minimum levels and the requisite skills not being regarded as scarce, drive down labour’s marketplace power (Boewe and Schulten, 2019; Kassem, 2023). It will be important to see how this develops within our current moment of lay-offs in the platform economy, resulting from unsustainable patterns of growth during the pandemic.

Marketplace and workplace power are closely related, as Amazon can weaken labour’s potential to disrupt the delivery of orders not only by shifting workers around in the warehouse, but also by shifting orders across its network (Apicella, 2021; Barthel, 2019; Kassem, 2023; Vgontzas, 2020). These power resources are further weakened by Amazon fragmenting its workforce, with temporary/seasonal labour possibly doubling the workforce during peak periods. While workers receive an hourly wage, their productivity rates measured in Units Per Hour act as a disciplining mechanism, especially for temporary workers who may risk losing their jobs if they mobilise their workplace and associational power (Apicella, 2021; Barthel, 2019; Delfanti, 2021; Kassem, 2023). This weakened marketplace power is therefore tied to their form of contract, as well as the decentralised network where Amazon pits workers within warehouses and across borders against each other, as seen in the case of German and Polish warehouses (Krähling, 2019; Owczarek and Chełstowska, 2018). Yet while its structuring conditions undermine labour’s power resources, Amazon claims that its

‘policies afford employees the freedom to form or join a labor organization or other lawful organization of their selection, collective bargaining, direct and indirect participation in workplace consultation structures, and access to redress mechanisms. We embed these policies across our business with direct employee involvement.’ (Amazon, 2022c)

With this in mind, I will now look in greater detail at Germany and the US, where the pandemic witnessed mobilisations of workplace power despite weak marketplace power (Kassem, 2022) and where workers navigated complex anti-union terrains in their pursuit of associational and institutional power.

### The continuing struggle for institutional power in Germany

Ver.di continued the longest labour struggle at Amazon during the pandemic, leveraging its workplace and associational power around health and safety in its pursuit of a collective agreement. Collective bargaining and co-determination through works councils and supervisory boards constitute the pillars of Germany’s ‘social partnership’ model. While this translated in the post-war years into growing wages and profits, these institutional powers declined and were partially eroded due to political-economic developments from the 1990s onwards. These developments included labour market deregulation and flexibilisation, deviations from collective bargaining agreements through so-called ‘opening clauses’, and a general decline in unions’ associational power – despite the rights to organise and unionise being enshrined in the constitution (Dribbusch et al., 2017; Vgontzas, 2020). As Amazon expanded in Germany, ver.di pushed for recognition of the collective bargaining agreement for the retail industry and not that of the logistics sector, as the former offered higher hourly wages. Amazon, however, views itself there as a logistics company and sets its wages accordingly (Apicella, 2021; Kassem, 2023; Vgontzas, 2020). This struggle continued during the pandemic. The increased potential for mobilisations did not, however, yield sufficient workplace and associational power to disrupt Amazon’s circulation line to achieve ver.di’s goal. Pre-existing weaknesses in these power resources, related to Amazon’s undermining structural conditions, overshadowed the pandemic context.

To start with, Amazon’s new hirings during the pandemic had repercussions on labour’s structural and associational powers. While hourly wages differ depending on a warehouse’s location, Amazon increased its hourly wages across the board for two months, adding €2 hazard pay. The varying purchasing power of the total hourly wage, at a time of initial economic insecurity, further decreased labour’s marketplace power. Moreover, workers may have risked going to work sick in order to benefit from this top-up (ver.di, 2020). With corona outbreaks reported in different warehouses, ver.di mobilised its associational and workplace power around health and safety concerns, with six warehouses coming out on strike at the end of June 2020 after 30–40 workers tested positive (Reuters, 2020). Crucial to labour’s growing pressure to get Amazon to the negotiating table, these powers were further mobilised during the peak season in 2020, a time of surging COVID-19 cases and a national lockdown which directed even more Christmas shoppers to Amazon. Against a backdrop of dozens of outbreaks in its warehouses and coinciding with Amazon’s reintroduction of hazard pay in November 2020, ver.di temporally and spatially coordinated its workplace power, calling up its associational power to take industrial action in six warehouses from the night of Monday, 21 December, to Christmas Eve. Though this was a peak time, Amazon claimed such mobilisations did not disrupt orders (Tagesschau, 2020), further underlining the difficulties in disrupting, let alone halting Amazon’s circulation line.

While ver.di continued to organise industrial action at different times during the pandemic, such as Prime Day, Black Friday, Christmas and Easter (see Tagesschau, 2021), the impact was undermined by Amazon’s general structural conditions predating the pandemic. A permanent contract gives workers the security to navigate their low marketplace power without jeopardising their jobs when mobilising their workplace and associational power. Workers, who under German industrial relations legislation cannot work for a company on a temporary contract for longer than two years, are thus more likely to join ver.di *if* and *after* they receive permanent contracts. While Amazon cannot legally block unionisation or industrial action in Germany, it may retaliate against organisers by moving them to exhausting, unpopular tasks like picking (Apicella, 2021; Apicella and Hildebrandt, 2019; Boewe and Schulten, 2019). The fragmentation caused by Amazon resorting to temporary and seasonal workers in peak periods has serious implications for their workplace and associational power, especially when considering that these hiring sprees overlap with the peak season when workers have the highest leverage (Barthel, 2019; Kassem, 2023). Non-permanent workers can then unwillingly act as strike-breakers if they continue working, a reality exacerbated by shifting workers across borders (Owczarek and Chełstowska, 2018). Taking into account the pace of seasonal hirings and Amazon’s expansion, it is difficult, yet vital, for ver.di to increase its workplace and associational power ‘in enough warehouses so that orders cannot be as easily rerouted’. This ‘requires that they coordinate with workers in other nodes who can ensure that management does not otherwise bypass these blockages’ (Vgontzas, 2020: 121).

Given the co-evolving relations of these powers, industrial action is crucial for gaining new union members, while being a union member paying monthly dues translates into a compensatory daily wage when striking. While older warehouses with more permanent and unionised workers, as found in Bad Hersfeld or Leipzig, are known to have stronger associational power and featured strikes before and during the pandemic (ver.di, 2022a), it is vital to expand shop-floor organising across the whole network. A recent case in point is Winsen, a relatively new warehouse near Hamburg which relies on migrant labour and is comparatively less unionised. Against a backdrop of inflation exceeding 10 per cent, Amazon decided unilaterally to raise hourly wages 3–7.4 per cent, though wages in Winsen were only raised 3 per cent (ver.di, 2022b). According to one interviewed worker, material interests united workers with dozens of nationalities and languages to strike in September–October 2022 and even unionise, thereby increasing both their workplace and associational power in an unprecedented manner. A Ghanian worker informed their fellow Ghanian workers, an Eritrean their fellow Eritreans, Syrians and Tunisians their Arab fellow workers and so forth, resulting in a snowball show of solidarity. Like others, the Winsen warehouse reflects larger racialised divisions of (manual) labour on the market, allowing Amazon to capitalise on its low entry barriers and migrants’ vulnerabilities in finding an employer prepared to accept their visa and residence status. Such workers are generally not familiar with the German industrial relations system, fear retaliation and, even if they wish to organise, may not be able to afford the union dues. Accounting for 1 per cent of their gross wages, these reduce the remittances sent to their families (Kassem, 2023). Migrant labour, like seasonal workers, are a key aspect of the labour struggle at Amazon, not just at the picket lines, but also as shop-floor stewards contributing to a wider sense of solidarity and representation. While multiple nationalities and languages may thus translate into a more fragmented workforce, this example demonstrates how lived experiences can also be crucial to growing labour’s resources.

While the German industrial relations system determines the labour battleground at Amazon, the company instrumentalises it to defend its dismissal of any necessity for a collective agreement. The centrepiece of the German Works Constitution Act is co-determination, with workers entitled to elect a works council when a company employs more than five workers. Indeed, Amazon works councils have wielded their institutional power to gain improvements in working conditions, minor wage increases, decentralised breakrooms or eliminate feedback talks in Bad Hersfeld (Apicella, 2021; Boewe and Schulten, 2019; Kassem, 2023; ver.di, 2022a; Vgontzas, 2020). While a warehouse’s degree of associational power increases the likelihood of a more unionised works council to represent workers’ interests, Amazon previously undermined this power, for instance by supporting more pro-employer candidates through printing their election flyers. Though Amazon cannot dodge works councils in Germany, it aims to fragment them as much as possible by having as few unionists as possible on them (Apicella, 2021; Boewe and Schulten, 2019, 2020). As works councils can be regarded as representatives of labour (Wright, 2000), Amazon indeed appears to use this form of institutional power to eliminate others. In a contradictory manner Amazon instrumentalises the existence of works councils as a space for dialogue and participation (Boewe and Schulten, 2020; Vgontzas, 2020), echoing its position of giving precedence to ‘direct employee involvement’ (Amazon, 2022c), thereby eliminating the need for additional associational and institutional power, i.e., a collective agreement. By default, this also implies a rejection of unions, given that they are necessary to bargain agreements, and thus also Amazon’s rejection of associational power.

The case of Germany reflects how Amazon’s undermining conditions have prevented workers from amassing sufficient leverage in support of their long-term goal of a collective bargaining agreement. While the pandemic presented additional potential for labour and ver.di’s continuing labour struggle, the obstacles faced by ver.di in organising workplace and associational power continued during the pandemic. This resulted in ver.di’s inability to successfully disrupt Amazon at a time of potentially high leverage. This demonstrates the importance of effectively bringing together labour’s power resources through shop-floor organising to boost solidarity among workers and their associational power. These are in turn vital for coordinating workplace power across the network to increase the potential for the disruptions needed – in the absence of regulation – to bring Amazon to the negotiating table.

### The pursuit of associational power in the US

Glancing across the Atlantic, the COVID-19 context presented a historical moment for workers in the US, triggering unprecedented mobilisations of workplace power around health and safety (Kassem, 2022). In certain warehouses, this brought about a push for associational power through unionisation which, if successful, legally obliges the employer to begin negotiations, thereby relating to their institutional power. Despite the US being Amazon’s oldest and largest market, the first attempt of US workers to unionise only occurred in 2001, at a Seattle call centre which Amazon subsequently shut down. This was followed in 2014 by another (unsuccessful) unionisation drive by technicians in Delaware (Del Rey, 2021). Unlike Germany, the US terrain, characterised by different industrial relations regimes, was largely not only strike-free, but also union-free until quite recently. Here again, Amazon continues to undermine labour’s power resources through its structural conditions but also through its union-busting activities.

With little marketplace leverage and needing to navigate complex terrains, the unprecedented expressions of workplace power during the pandemic were very important. Amazon’s hourly wages in its US warehouses, start at over US$18 and are thus more than double the federal minimum wage (US$7.25). These were similarly topped up by US$2 hazard pay during the pandemic, accompanied by health insurance. Given labour’s weak marketplace power and no unionised associational power, the first workplace mobilisation can only be traced back to 2018–2019 when Minnesota’s for the most part East African warehouse workers walked off the job. Reproducing a ‘racialized division of warehouse labor, with white men disproportionately employed as managers, and people of color employed as warehouse associates’ (Reese, 2020: 104), these workers showed solidarity precisely through their racialised subjectivities and realities. Supported by the East African workers-led community centre, the *Awood Center*, workers mobilised around the shared goals of reducing workloads during the fasting month of Ramadan and the Eid festivals (UNI and ITUC, 2019). While such mobilisations did not trigger nationwide action, this was to occur in 2020 against a backdrop of coronavirus exposure and risks. Walkouts unfolded across the US, ranging from Minnesota and Chicago to California and New York (O’Brien, 2022; Paul, 2020; UNI Global Union, 2020). A prominent case was the Staten Island walkout organised in March 2020 by Christian Smalls, who was then fired on grounds of violating his quarantine and labelled by Amazon as ‘not smart or articulate’ to be made the ‘face of the entire union/organizing movement’ (Blest, 2020). While these industrial actions did not halt or severely disrupt the circulation line, the pandemic triggered, even in the absence of associational power, unprecedented mobilisations of workplace power in Amazon’s most important market (Kassem, 2022).

Given labour’s co-evolving power resources, this historical moment was additionally accompanied in some warehouses by an unprecedented pursuit of associational power. While works councils are as yet non-existent in US warehouses and while unions like Teamsters and RWDSU and movements like Amazonians United have generally supported the labour struggle, until April 2022 unionised associational power was de facto absent. Under the 1935 National Labor Relations Act (NLRA), workers must first demonstrate to the National Labor Relations Board (NLRB) through ballots or sign cards that at least 30 per cent of a company’s workforce wish to unionise for an election to take place. If a majority is achieved, the NLRB certifies the union ‘as a representative for collective bargaining’, with the employer legally required to not just recognise the union but also begin collective bargaining. In the event of a majority in the prior step, the employer can voluntarily recognise the union without an election (National Labor Relations Board, n.d.). While the NLRA was intended to facilitate and safeguard unionisation and collective bargaining, the billion-dollar anti-union lobby and Amazon’s union-busting tactics have sought to prevent such associational and institutional powers from forming (Boewe and Schulten, 2019). In a time of increased workplace and associational mobilisations in the US, Amazon spent over US$4m on anti-union consultants in 2021 alone (Del Rey, 2022; Palmer, 2022).

The pursuit of associational power within the context of the pandemic and the Black Lives Matter movement was triggered by Darryl Richardson, a warehouse worker in Bessemer, Alabama. This first union drive of any size in an Amazon warehouse, here via the RWDSU, was termed by RWDSU’s president as ‘as much a civil rights struggle as it is a labour struggle’ (Del Rey, 2021). While the racialised realities of Bessemer’s predominantly black workforce can be a key factor for solidarity and mobilisation, Bessemer workers were faced with weakened marketplace power in a state with no minimum wage and a fragmented workforce in a warehouse recently opened in March 2020. On top of these disadvantages came the context of the Deep South in the US. According to interviewees of a study, this was a context in which Amazon’s ‘policing power’ meant that

‘[p]olice frequently supplement internal private security to resolve workplace disputes, and routine police surveillance combined with Amazon’s own internal surveillance via digital and other means has led some Black workers to describe the Amazon fulfillment center as a jail and/or a modern plantation that makes them feel like “slaves”.’ (Lee et al., 2022)

Such ongoing repression along with Amazon’s generally undermining structural conditions go against any mobilisation of labour’s power resources, leaving workers feeling vulnerable. Nevertheless, the Bessemer workers achieved the 30 per cent threshold necessary for a union election. Considering that under US industrial relations a successful union drive would impact both associational and institutional power, Amazon waged an anti-union campaign leaving ‘no stone unturned’ (RWDSU, 2021). Overlapping with the peak season, the hiring of thousands of new workers required new organising efforts (Todd, 2021), while Amazon resorted to such actions as hiring anti-union consultants for US$10,000 a day and allegedly requesting shortened traffic-light intervals outside the warehouse to prevent unionists approaching workers. In addition to installing an outdoor Postal Service ballot-box, which the NLRB deemed to violate labour law (Bethea, 2021; Greene, 2021), Amazon went as far as to organise mandatory ‘captive audience meetings’. These framed unions as being against workers’ class interests, arguing that unionisation meant slashing benefits, possible warehouse closures and obligatory union dues – in fact prohibited in Alabama, a ‘right to work state’ (Del Rey, 2021). Though Amazon’s actions resulted in a revote called by the NLRB, the labour struggle at Bessemer continues within this context of the Deep South, weakened marketplace power and Amazon’s union-busting. These all hold severe repercussions for labour’s power resources and shop-floor organisation.

While Bessemer set a precedent with its first election, JFK8 marks the first successful union drive and, as of April 2022, the first unionised associational power in Amazon’s US history. Despite different local contexts, weak marketplace power and Amazon’s undermining of structural conditions, the workers of this racialised manual workforce voted for the Amazon Labor Union (ALU). This ‘independent, grassroots, worker-led union’ founded in 2021 by fired worker Smalls and current worker Derrick Palmer, both of whom know the ‘ins and outs of the company’, organised workers and built solidarity by for instance setting up a tent at the public bus stop outside the warehouse, offering food and informing workers across shifts (Amazon Labor Union, n.d.; New York Times, 2022). Amazon resorted to many of the tactics employed in Bessemer, like mandatory captive meetings and hiring a polling and consultancy firm, in this case the Global Strategy Group, to create and distribute anti-union material (Del Rey, 2022; Palmer, 2022). A trigger for the formation of labour solidarity came after Smalls and other organisers were arrested by the police for trespassing while distributing food to workers (Hsu, 2022; New York Times, 2022). Despite this larger anti-union context, ALU’s grass-root approach in connecting with workers and building solidarity was a key factor in gaining the associational power manifested at this warehouse, the same one where Smalls initially mobilised in 2020. Workplace power at this warehouse thus organically helped establish the ALU and its fight for associational power. While Amazon regards the ALU as a ‘third party’ in the labour struggle (Hsu, 2022), refuses to recognise it and has filed additional objections with the NLRB, the NLRB has reacted by certifying the ALU as representing workers at JFK8 (Scheiber, 2022). As the ALU seeks to increase the hourly wage to at least US$30, eliminate mandatory overtime in non-peak times and push for longer breaks (Del Rey, 2022), it remains to be seen how this associational power translates into institutional power and bargaining.

In these complex national and local terrains where elections have not always been successful, the above-mentioned examples of Bessemer and JFK8 demonstrate, among other things, two crucial points. First, the pandemic played a crucial role in enabling workers to pursue their workplace and associational power in an unprecedented manner. The victory at JFK8, a warehouse with over 8000 workers, has been regarded as the largest unionisation victory since GM and 1930s automotive industry (New York Times, 2022). While this constitutes a milestone for Amazon workers and underlines the potential of grass-root organising, ongoing battles for associational power elsewhere shed light on the second crucial point: the sheer range of Amazon’s union-busting activities that, along with its undermining structural conditions, aim to quash the labour movement and its ability to create sufficient pressure to successfully disrupt Amazon’s most important market.

## Discussion and conclusion

COVID-19 and co-evolving political-economic conditions and regulations initially resulted in Amazon’s exponential growth and the concomitant intensification of labour struggles for its essential workers. The cases of Germany and the US shed light on how workers, despite low marketplace power, were able to mobilise their workplace power around health and safety concerns (Kassem, 2022). While these cases demonstrate the renewed potential for organising labour, they also demonstrate how Amazon’s structural conditions and anti-union position persist as obstacles in the way of workers disrupting Amazon’s business and fully cultivating their associational and institutional power.

Both national cases, despite their varying industrial relations settings, illustrate how Amazon is able to maintain its position of rejecting unions as negotiating partners, regarding them as third parties unnecessary for dialogue. While Amazon cannot legally fight works councils in Germany or the existence of ver.di, it uses the former to eliminate the latter and its goal of a collective bargaining agreement. As ver.di continues to mobilise, its labour struggle appears to be taking on a character of continuation rather than achieving fundamental change, even in the context of COVID-19. By contrast, the pandemic acted as a catalyst in the US for unprecedented mobilisations from workplace to associational power. While the latter has as of yet been unsuccessful in the majority of cases, it has triggered a key process of pursuing unionisation in a context where Amazon has ramped up its union-busting activities. Despite the different industrial relations settings in the two countries, Amazon is able to dodge negotiations, and therefore labour’s associational power in the form of unions, unless it is legally obliged to negotiate. Amazon continues to ‘exploit the leeway allowed by national laws’ and instrumentalise their contexts (Boewe and Schulten, 2020: 211).

Analysing labour’s co-evolving power resources within their different contexts demonstrates how, in a moment of potentially higher leverage, Amazon’s most important markets could not put pressure on Amazon. The persistence of structural obstacles and conditions that predate the pandemic underline the continuing need for shop-floor and grass-root organising to boost workplace and associational power across the Amazon network. This also means further account being taken of the various compositions of the workforce. While this article has only touched on the racialised dimensions of the workforces, it is crucial to continue research efforts examining how these, along with gendered dimensions, systematically fragment workforces while binding workers. The stronger the local mobilisations of workplace and associational power are, the greater the potential for national or even transnational power to put pressure on Amazon. However, if even its two most important markets are unable to generate sufficient pressure to get Amazon to the negotiating table, then labour’s ongoing struggle over its power resources needs to be supported politically by regulatory frameworks defining the bounds of Amazon’s activities. As these cases demonstrate, if industrial relations systems do not obligate the company to recognise or negotiate with unions, Amazon, backed by its multi-million-dollar efforts, will instrumentalise the terrain to undermine, and if possible quash, labour organisation and the labour movement. As it becomes increasingly difficult for platforms such as Amazon to sustain the exponential growth they experienced during the pandemic, forcing them to lay off thousands of workers, it will be even more important to continue examining how labour’s power resources evolve but also how to further support these labour struggles.
